# Transgranular liquation cracking of grains in the semi-solid state

**DOI:** 10.1038/ncomms9300

**Published:** 2015-09-10

**Authors:** S. Karagadde, P. D. Lee, B. Cai, J. L. Fife, M. A. Azeem, K. M. Kareh, C. Puncreobutr, D. Tsivoulas, T. Connolley, R. C. Atwood

**Affiliations:** 1 Manchester X-ray Imaging Facility, School of Materials, The University of Manchester, Manchester M13 9PL, UK; 2 Research Complex at Harwell, Harwell Science & Innovation Campus, Didcot OX11 0FA, UK; 3 Swiss Light Source, Paul Scherrer Institut, Villigen PSI 5232, Switzerland; 4 Department of Materials, Imperial College London, London SW7 2AZ, UK; 5 Department of Metallurgical Engineering, Chulalongkorn University, Bangkok 10330, Thailand; 6 Diamond Light Source Ltd, Harwell Science & Innovation Campus, Didcot OX11 0DE, UK

## Abstract

Grain refinement via semi-solid deformation is desired to obtain superior mechanical properties of cast components. Using quantitative *in situ* synchrotron X-ray tomographic microscopy, we show an additional mechanism for the reduction of grain size, via liquation assisted transgranular cracking of semi-solid globular microstructures. Here we perform localized indentation of Al-15wt.%Cu globular microstructures, with an average grain size of ∼480 μm, at 555 °C (74% solid fraction). Although transgranular fracture has been observed in brittle materials, our results show transgranular fracture can also occur in metallic alloys in semi-solid state. This transgranular liquation cracking (TLC) occurs at very low contact stresses (between 1.1 and 38 MPa). With increasing strain, TLC continues to refine the size of the microstructure until the grain distribution reaches log-normal packing. The results demonstrate that this refinement, previously attributed to fragmentation of secondary arms by melt-shearing, is also controlled by an additional TLC mechanism.

## Abstract (web summary)

To obtain superior mechanical properties of cast components, grain refinement via semi-solid deformation is desirable. Here, the authors use in situ X-ray tomography to study the response of an alloy to indentation, and present a new mechanism of transgranular liquation cracking.

## Graphical abstract

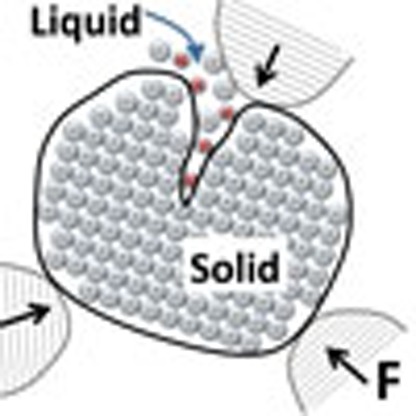

A number of *in situ* experimental^1^,^2^,^3^,^4^ and modelling^5^ studies have recently reported how partially solidified metals can deform with the characteristics of a granular material^1^,^6^,^7^; specifically, they form defects between the grains via pores/damage occurring in the intergranular liquid^1^,^2^,^3^. However, a less-frequently reported observation in non-metallic semi-solid systems is transgranular cracking, which has been observed in low-temperature brittle metals^8^,^9^, nano-crystals^10^, graphene^11^ and other brittle materials (for example, glass beads^12^ and rocks^13^,^14^,^15^). This failure mode has also been reported in a few high-temperature systems, such as magmas^16^,^17^, using post-mortem analyses. Although there have been a few cases of transgranular failure reported in completely solid metals during fatigue at high temperature^18^, such a mechanism has not been reported in semi-solid metals, where the primary (solid) phase is expected to be very ductile. Similarly, the process of liquation cracking, or cracking along the liquid channels between grains, also known as intergranular failure, is observed in heat affected zones during welding of aluminium alloys^19^, austenitic steels^20^ and superalloys^21^. However, it has not been reported to occur within grains, typically due to the lack of pre-existing liquid channels.

Semi-solid microstructural response to the imposed deformation is important in advanced alloys because they are subject to deformation due to shrinkage forces and thermal contraction during conventional processing or when shear forces are applied^5^,^22^,^23^,^24^. In these cases, a temperature window is encountered where solid grains and intergranular liquid co-exist; that is, the material is semi-solid but capable of transmitting load^25^,^26^. The microstructural response to these forces is known to strongly depend on the grain size and morphology of the material^27^, which is important for thixoforming and other forming processes^28^,^29^. The reduction in grain size during semi-solid processing has been primarily attributed to fragmentation of secondary arms^25^,^30^,^31^. During shearing of semi-solid melts containing equiaxed dendrites, secondary arms detach from the primaries, and subsequently coarsen to form globular microstructures. This fragmentation process has been hypothesized to occur by formation of high-angle grain boundaries (large plastic deformations), and eventual detachment^30^, in addition to remelting and pinch-off^25^,^32^. Doherty *et al.*^30^ also hypothesise that these high-angle grain boundaries might be subjected to wetting, further reducing the energy required to break these secondary arms. Likewise in some brittle materials, such as rock salts and Cu-based alloys, transgranular cracking in single crystals has been accelerated by the presence of a liquid phase (saturated solution) inducing a stress-corrosion cracking-like mechanism^14^,^33^.

In this study, we investigate the micro-mechanical response and failure mechanisms of a semi-solid granular material during indentation using *in situ* synchrotron X-ray tomography. Capturing the process at temperature allows us to quantify the motion, deformation and failure of large globular grains in a semi-solid alloy. Indentation of Al-15 wt.%Cu semi-solid globular microstructures was performed using fast high-resolution X-ray tomographic microscopy at the I12 beamline at the Diamond Light Source, UK^34^. Although indentation is typically employed on solids, the purpose of using an indenter in this study was to obtain a localized deformation, preferably by pushing a single grain to perturb and stress the granular system.

## Results

### *In situ* observation

**Figure 1:**
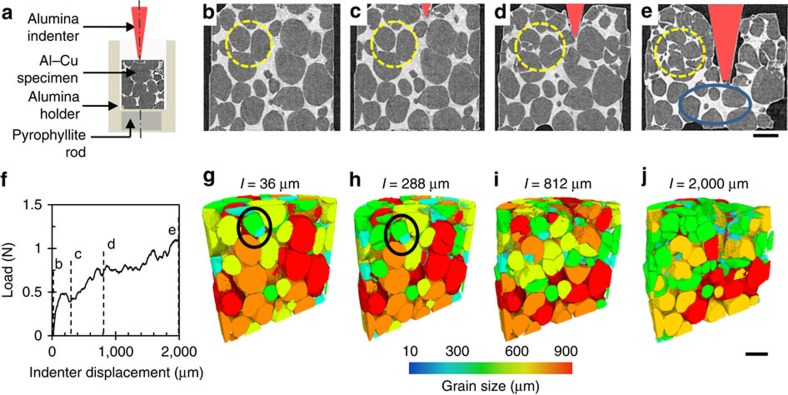
Semi-solid indentation of globular microstructures. (**a**) schematic of the set-up, (**b–d**) sequence of 2D longitudinal slices, with the indenter coloured red, showing the onset and development of transgranular cracking of globular microstructures at 555 °C during indentation with a speed of 2 μm s^−1^ (corresponding to the instances marked in **f**). (**e**) The final room temperature scan. (**f**) The load measurement (averaged). (**g–j**) The corresponding 3D sectional views of segmented solid grains (coloured by size). ‘*I*' denotes the corresponding indenter position. The yellow dashed circles in **b–e** and the black circles in **g** and **h** highlight region of interest where transgranular fracture of seemingly ductile grains is occurring. The blue circle indicates the region of apparent increase in liquid after indentation. Scale bar, 500 μm.

The *in situ* tomographic observations of indentation into a semi-solid alloy are shown in Fig. 1. The motion of the indenter (red, 2 μm s^−1^) and granular flow of the solid α-Al grains (light grey) in the copper-enriched liquid (white) is captured at four time points (Fig. 1b–e; Supplementary Movie 1). Unlike any prior studies on semi-solids, these results capture the first instance of transgranular fracture of ductile grains (Fig. 1d, region of interest marked with a yellow circle). This is shown in three-dimension (3D) in Fig. 1g–j. At the beginning of the deformation (Fig. 1c,h), grains near the indenter show granular flow before being constrained by their neighbours. This is followed by significant cracking of the highlighted grains in the region of interest as well as those around the indenter (Fig. 1d,i). Several other grains undergo cracking on continued deformation as evident from the final scan at room temperature (Fig. 1e,j). Note that the cracks are liquid filled. The load steadily increases during indentation via a series of small jumps (Fig. 1f); most likely due to an increase in local densification of the solid with the load relaxing each time a grain fragments. Similar results were observed for other indenter displacement rates of 0.5 and 10 μm s^−1^, with prominent regions of cracking below the indenter (Supplementary Fig. 1).

The region of highly fractured grains is localized near the indenter, with the grains in the bottom half only undergoing granular motion (2 μm s^−1^; Fig. 1e), suggesting that a localized force chain^35^,^36^ forms where a critical cracking stress is exceeded. Similarly, in the other two cases (Supplementary Fig. 1), the transgranular cracking is localized into a single constrained region. As grains crack, the fragments rearrange through granular flow accommodating the applied strain for a short time, until the fragments are pinned again, with contact stresses rising sufficiently to crack the grains into increasingly smaller fragments. This fragmentation causes localized densification of the solid phase, forcing liquid into the regions with lower load and no fragmentation. This is quantitatively compared in Fig. 1e where the yellow fragmented region has a solid fraction of 78% and the blue circled region has a solid fraction of 65%. Scanning electron microscopy and electron backscatter diffraction (EBSD) images of the initial microstructure and final cracked microstructure (Supplementary Fig. 2; Supplementary Note 1) clearly show transgranular fracture. Interestingly, even when examining such early works as those of Doherty *et al.*^30^ and Flemings^25^ on semi-solid deformation, similar features where a globular grain has split into two hemispherical halves can be observed; however, the present study is the first to provide an explanation for these features.

### Estimation of limiting contact stress

**Figure 2:**
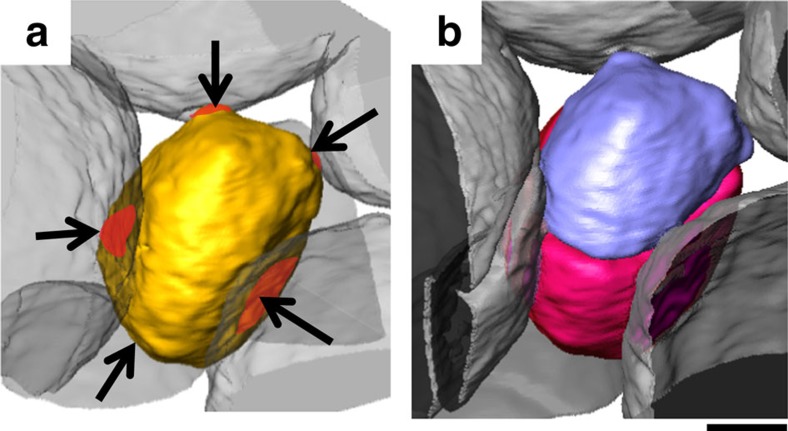
3D visualization of transgranular liquation cracking. 3D rendering of a grain (shown inside the black circle in Fig. 1g–h) cracking into two during indentation: (**a**) initial arrangement at *I*=0 μm with the contact areas in the field of view coloured red and (**b**) cracked morphology at *I*=144 μm. Scale bar, 200 μm.

Figure 2a shows one of the first fractured grains near the indenter tip (rendered in yellow) before cracking; its immediate contacting neighbours are coloured grey and their common contact areas are coloured red. After an indenter displacement of 144 μm, the grain cracks into two pieces (Fig. 2b, purple and pink). The observations in Figs 1 and 2 (Supplementary Figs 1 and 2) show that any grain in the sample can potentially crack if it is constrained and loaded. To estimate the contact stress responsible for cracking, we consider a layer (or bed) of constrained grains (within a volume of an average grain's height), to which the applied load is transmitted (shown schematically in Supplementary Fig. 3). Let ‘*F*' be the load from the indenter (transmitted to any single grain within a constrained layer) linked through a force chain. The areas normal to the applied load (marked in blue in Supplementary Fig. 3) are likely to transfer the load directly. An estimate of stress transmitted through these normal areas is given by 
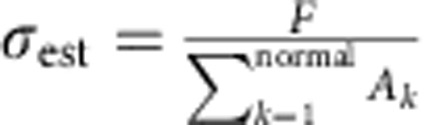
. We can bound this estimate with a lower bound stress, calculated by assuming *F* is transmitted through all possible contact areas, with 
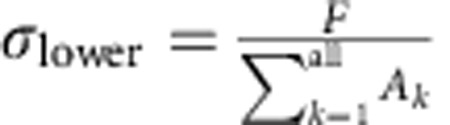
, and an upper bound using just a single contact^12^, 
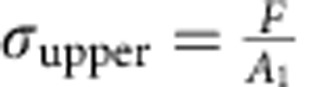
. The contact areas were calculated directly from the 3D tomographic images captured immediately before the grain cracked. From this, the estimated stress, and lower and upper bounds of the load when this grain cracked (*F*=0.49±0.1) are: 11.8±2.5, 1.1 and 37.7 MPa, respectively. The bounds also enclose the measured flow stress of ∼10 MPa for Al-3 wt.%Cu material at 555 °C in ref. 5. The variation in lower and upper bounds is large, and the localized force chains are difficult to determine experimentally. Although the contact stresses between grains have previously been estimated via force chains and discrete element simulations idealized systems^35^,^36^,^37^, such computational techniques have yet to include the multi-physics approaches required to simulate remelting and liquation processes.

### Grain size distribution

**Figure 3:**
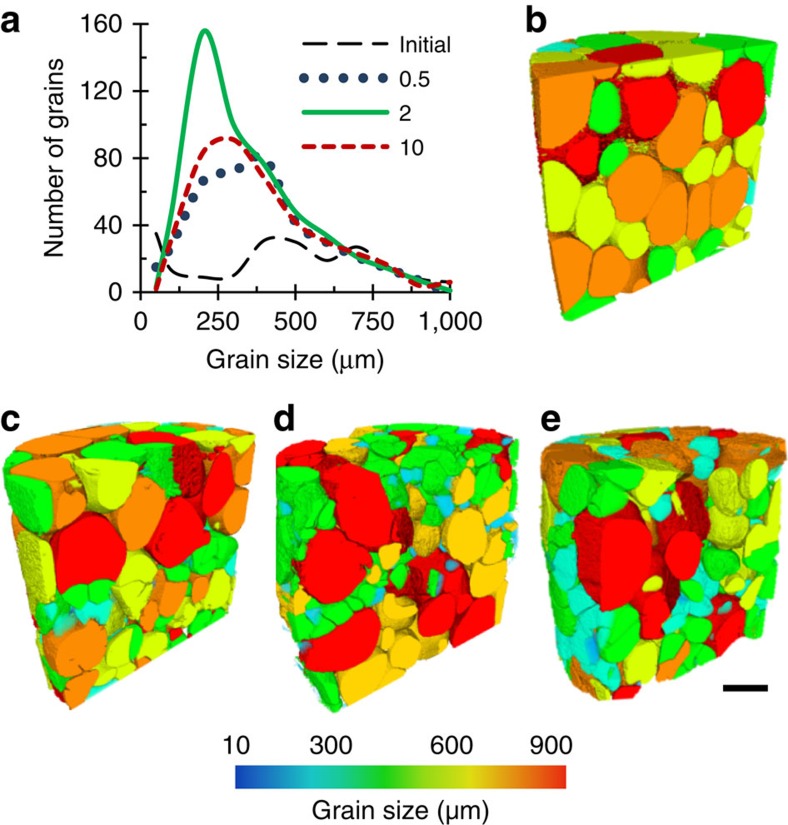
Log-normal distribution of cracked grains. (**a**) Grain size distribution of initial and final Al–Cu microstructures after semi-solid indentation at various indentation speeds (units: μm s^−1^). 3D rendering of solid grains, coloured by size, at (**b**) the initial state for the 0.5 μm s^−1^ indentation speed and at the final states for the (**c**) 0.5, (**d**) 2 and (**e**) 10 μm s^−1^ indentation speeds, respectively. Scale bar, 750 μm.

The initial grain size distribution for the 2 μm s^−1^ case is plotted in Fig. 3a (note the initial distribution of grains is near identical for all samples since they were all machined from one starting block), together with the final size distribution at the end of the indentation sequence for the three indenter velocities. Interestingly, for all speeds, indentation causes the grains to fracture such that they form log-normal distributions. Such a log-normal particle distribution is one of the optimal distributions for obtaining maximum packing density^12^,^38^. This implies that the system is responding to the imposed deformation by naturally increasing its packing density to best distribute the load. The 3D sectional views of the final microstructures at indenter displacement rates of 0.5 and 10 μm s^−1^ (Fig. 3c,e, respectively), show that the localized force chain and associated cracking occurred directly below the indenter. In all cases, the mode of the grain size halves on deformation, and the number of grains doubles (as listed in Table 1), but the greatest cracking and size reduction happens at 2 μm s^−1^ (Fig. 3d), where the first force chain forms to the sidewall. This observation confirms the stochastic behaviour of granular flow, even when constrained and subject to transgranular failure. It is important to note that these measurements are limited by the pixel size (4 μm per pixel) of the imaging setup from I12 used in this study, whilst scanning electron microscopy images show cracks and liquid films that have thicknesses less than this^39^ (Supplementary Fig. 2).

### Role of solid volume fraction

**Figure 4:**
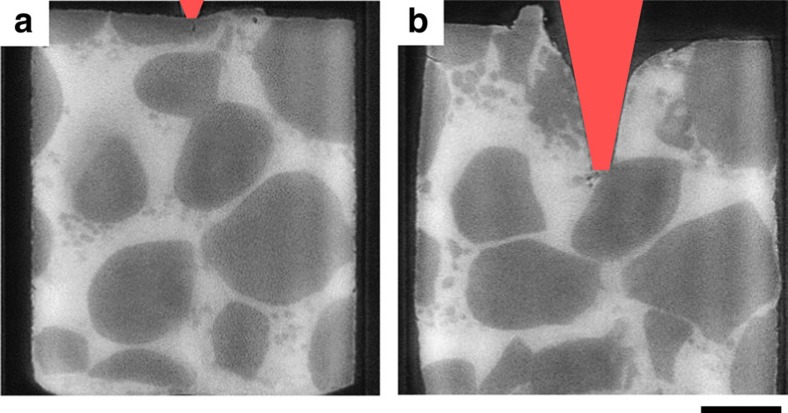
Influence of solid fraction. Longitudinal slices of semi-solid indentation of large globular Al–Cu grains at 570 °C: (62% solid volume fraction, average grain size: ∼450 μm): (**a**) before and (**b**) after 1-mm indentation at 0.5 μm s^−1^. No cracking was observed unlike with the 73% solid volume fraction sample. (Note: fine light grey patches are assumed to be newly nucleated grains due to thermal fluctuations; scale bar 400 μm).

To determine the dependence of cracking on the amount of liquid between grains, indentation experiments were performed at a higher temperature (570±5 °C) with a speed of 0.5 μm s^−1^, where the solid volume fraction was 62±2% (as compared with 73% fraction solid for the previous experiments). With the increased liquid fraction (from 27% to almost 40%), no cracking was observed; see Fig. 4 and Supplementary Table 1 for further experimental details. Much more granular motion can be observed, with the grains freely translating and rotating to accommodate deformation^1^. From this data, it appears that the propensity of cracking is directly influenced by the solid volume fraction. This is also consistent with the observations shown in Fig. 1e, where increased packing in the fragmented region was observed. Furthermore, the occurrence of cracking is independent of the indenter shape (Supplementary Fig. 4) and the deformation rate within the observed range of deformation speeds (0.5–10 μm s^−1^). Hence, we can hypothesise that there is a minimum solid fraction above which the grains contact sufficiently to form force chains that constrain motion, and this leads to transgranular fracture.

## Discussion

These experimental findings pose important questions about how and why transgranular cracking can occur so readily in the single-crystal primary phase (α-Al) globular grains of an alloy in the semi-solid state. Prior studies have only reported cracking (and other defects such as damage voids) to occur between grains, or intergranularly^1^,^2^,^3^,^4^,^5^,^6^,^7^. Further, liquation cracking during welding^20^ and liquid metal embrittlement^40^ are also assumed to occur intergranularly. This is the first time transgranular liquation cracking (TLC) has been observed *in situ* and quantified in semi-solid alloys, similar to transgranular cracking in brittle materials.

**Figure 5:**
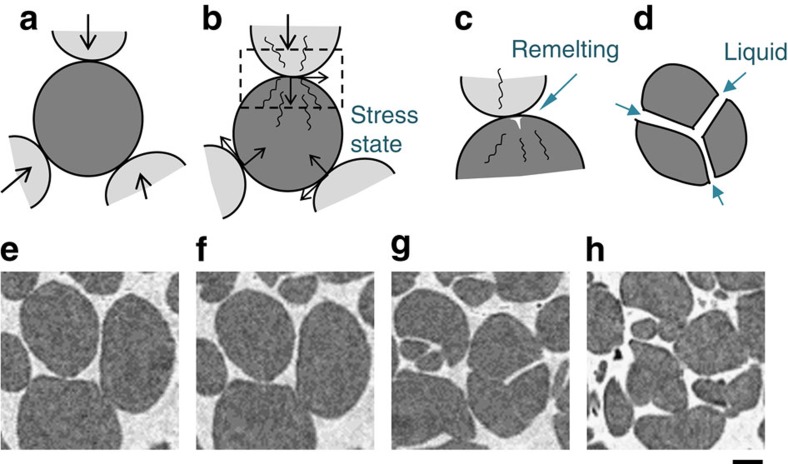
Mechanism of transgranular liquation cracking. (**a–d**) 2D schematic of the four stages: (**a**) motion and pinning of grains resulting in compressive and shear loads; (**b**) straining and dislocation movement under a stress state; (**c**) possible remelting and interface perturbation (shown for only one contact); and (**d**) crack growth from the surface of the grain accelerated by liquid entrainment. (**e**–**h**) Region of interest from Fig. 1b–e (yellow dotted circle) where transgranular fracture of single-crystal grains is occurring. Scale bar, 150 μm.

On the basis of the experimental observations, we hypothesize that the cracking happens via a combination of mechanisms in a series of stages, shown schematically in Fig. 5a–d. As a semi-solid globular alloy is compressed under constraint and the fraction of liquid is low, intergranular liquid will flow to regions of lower pressure^2^,^22^,^23^,^41^ and grains will contact each other forming a force chain. Note that each grain is a near-perfect single crystal but has a random crystallographic orientation (Fig. 5a; Supplementary Fig. 2). Once the grains become pinned, they will apply compressive and shear loads at a range of misorientations depending on the orientation of the grain. A number of neighbours will apply small but multiple contacts around the primary α-grain (Fig. 5b). This leads to localized elastic and small plastic strains (Fig. 5c), generating dislocations that move to the surface quickly (dislocation mobility increases at elevated temperatures). The dislocations are likely to pin and interlock, as well as cause roughness at the surface and a localized increase in free energy, which results in localized remelting. The intergranular liquid is then drawn into the crack, causing TLC (Fig. 5d). Note that the coarsening process can lead to an occasional formation of small liquid pockets^25^ (<10 μm size), which act as potential stress concentrators deflecting the crack (curved crack in Supplementary Fig. 2b). Similar to stress- and liquid-assisted cracking of rock salts^42^ and Cu-based alloys^33^, the interdendritic liquid surrounding these grains acts as an interface destabiliser, inducing a stress-corrosion type acceleration of the crack (compressive stresses required for fracture range from 3 to 100 MPa (refs 14, 43)). The proposed mechanism can also be correlated to fragmentation of secondary arms during melt shearing^25^,^30^, where forced melt flow induces large plastic deformation, causing remelting^25^ and wetting^31^-assisted detachment. We also see that the cracking occurs recursively (Supplementary Fig. 4) until no further contacts with required stress levels remain, that is, a log-normal distribution in grain size is reached.

The four stages during TLC are therefore hypothesized (Fig. 5): motion and pinning of grains resulting in compressive and shear loads (Fig. 5a); dislocation generation, leading to surface perturbations and internal preferential crack paths^44^ (Fig. 5b); remelting^25^,^32^ at the surface perturbation^45^ (Fig. 5c); and crack growth from the surface of the grain accelerated by liquation (Fig. 5d). Although drawn schematically in Fig. 5a–d, we observe these same stages in our experiments, as shown in Fig. 5e–h from a zoomed-in region circled in Fig. 1b–e, clearly demonstrating the power of *in situ* synchrotron experiments to inform and quantify new mechanisms.

In summary, using *in situ* synchrotron X-ray tomographic microscopy, we have shown for the first time that, in addition to known fragmentation mechanisms, TLC of grains contributes to grain size reduction in semi-solid alloys with high solid volume fractions. Localized deformation comparable to an average grain size on a constrained specimen is found to initiate cracks. Furthermore, liquation is observed to assist the propagation of cracks and the eventual granular separation. This fragmentation of primary α-grains leads to final microstructures with log-normal size distributions, providing the highest packing density and optimal load distributions for the microstructures. The study has presented a quantified hypothesis of a new mechanism to achieve grain refinement that is relevant to materials processing, magmatic flows and oil/mineral extraction.

## Methods

### Thermomechanical setup

A bespoke thermal-mechanical setup was used to perform the semi-solid indentation. The apparatus consisted of a mechanical testing rig (P2R)^1^,^2^,^46^ and a loop-feedback controlled resistance furnace with an X-ray transparent window^1^,^2^,^47^. The samples were previously prepared by heat-treating the alloy in the semi-solid state for 23 days at 555 °C (7 °C above the eutectic temperature)^1^. A 3-mm diameter, 3-mm long (∅ 3 × 3 mm) cylindrical specimen was placed at the centre of an alumina holder (Fig. 1a) in preparation for tomographic imaging. This specimen size ensured sufficient number of globular grains within the sample, without compromising the pixel resolution. The specimen was heated at a rate of 40 °C min^−1^ to 555±2 °C and subsequently held isothermally for 10 min. At this temperature, the sample volume had a solid volume fraction of 73±2% (as determined by image processing). Each sample was then indented using a conical alumina indenter (15^o^ cone angle) at displacement rates of 0.5, 2 and 10 μm s^−1^, or equivalently, with strain rates of 6 × 10^−4^, 1 × 10^−3^ and 5 × 10^−3^ s^−1^, respectively^48^. Indentation experiments were also performed on smaller ∅1.8 × 2-mm cylindrical specimens at the TOMCAT beamline of Swiss Light Source^49^ (Paul Scherrer Institut, Villigen Switzerland), providing a range of liquid volume fractions. The mechanical setup was similar but the heating was performed using a laser-based heating system^50^ instead of a resistance furnace.

### Data acquisition and image processing

During the experiment at the I12 beamline of Diamond Light Source, 2 sets of 9 tomographic scans at 9-s intervals were acquired, with a 5-min interval in between each set of scans to offload the data from the camera. Each scan consisted of 900 projections, acquired using a 53-keV monochromatic X-ray beam. The camera consisted of a single-crystal cadmium tungstate scintillator, lens coupled with a Vision Research Miro 310M camera, giving a 4-μm pixel size. Each 3D scan was reconstructed using filtered backprojection^51^ to produce a 1,200 × 1,200 × 800 voxel volume per scan. Each 3D volume was filtered with median and anisotropic diffusion filters to reduce the noise, and then solid grains were segmented using a watershed-separation algorithm in Avizo (FEI VSG, France).

## 

## Additional information

**How to cite this article:** Karagadde, S. *et al.* Transgranular liquation cracking of grains in the semi-solid state. *Nat. Commun.* 6:8300 doi: 10.1038/ncomms9300 (2015).

## Supplementary Material

Supplementary Information Supplementary Figures 1-4, Supplementary Table 1 and Supplementary Note 1

Supplementary Movie 1 Isothermal indentation of semi-solid globular microstructures of Al-15wt%Cu alloy at 74% fraction solid. The indenter is moving at a constant speed of 2 μm s^-1^. Longitudinal slices from the 3D reconstructed data are shown.

## Figures and Tables

**Table 1:** Comparison of cracking data for different deformation speeds.

**Indentation**		**Solid fraction (%) (at 555 **°**C)**	**Before indentation**			**After indentation**		
**Speed (μm s**^**−1**^)	**Depth (mm)**		**Number of grains**	**Drain diameter (μm)**		**Number of grains**	**Grain diameter (μm)**	
				**Mean**	**s.d.**		**Mode**	**s.d. (log-normal)**
0.5	0.8	73.7±2	159	484	234	383	380	151, 242
2	2	73.4±2	162	489	253	511	205	126, 232
10	2	73.5±2	168	481	226	364	295	173, 384

## References

[b1] Kareh K. M., Lee P. D., Atwood R. C., Connolley T. & Gourlay C. M. Revealing the micromechanisms behind semi-solid metal deformation with time-resolved X-ray tomography. Nat. Commun. 5, 4464 (2014). 25034408 10.1038/ncomms5464 PMC4109016

[b2] Cai B. *et al.* *In situ* synchrotron tomographic quantification of granular and intragranular deformation during semi-solid compression of an equiaxed dendritic Al–Cu alloy. Acta Mater. 76, 371–380 (2014).

[b3] Terzi S. *et al.* *In situ* X-ray tomography observation of inhomogeneous deformation in semi-solid aluminium alloys. Scr. Mater. 61, 449–452 (2009).

[b4] Nagira T. *et al.* Direct observation of deformation in semi-solid carbon steel. Scr. Mater. 64, 1129–1132 (2011).

[b5] Fuloria D. & Lee P. D. An X-ray microtomographic and finite element modeling approach for the prediction of semi-solid deformation behaviour in Al – Cu alloys. Acta Mater. 57, 5554–5562 (2009).

[b6] Gourlay C. M. & Dahle A. K. Dilatant shear bands in solidifying metals. Nature 445, 70–73 (2007). 17203058 10.1038/nature05426

[b7] Fonseca J., O'Sullivan C., Nagira T., Yasuda H. & Gourlay C. M. *In situ* study of granular micromechanics in semi-solid carbon steels. Acta Mater. 61, 4169–4179 (2013).

[b8] Hasegawa T. & Ilschner B. High-temperature transgranular fracture in an austenitic stainless steel. Scr. Metall. 17, 523–527 (1983).

[b9] Liu S., Liu D. & Liu S. Transgranular fracture in low temperature brittle fracture of high nitrogen austenitic steel. J. Mater. Sci. 42, 7514–7519 (2007).

[b10] Gu P. & Dao M. Size-dependent deformation in nanograins and nanotwins. Appl. Phys. Lett. 102, 091904 (2013).

[b11] Zhang P. *et al.* Fracture toughness of graphene. Nat. Commun. 5, 3782 (2014). 24777167 10.1038/ncomms4782

[b12] Walker W. J. Jr. Persistence of granular structure during compaction processes. KONA Powder Part. J 21, 133–142 (2003).

[b13] Eberhardt E., Stead D., Stimpson B. & Read R. S. Changes in acoustic event properties with progressive fracture damage. Int. J. Rock Mech. Min. Sci. 34, e1–71.e12 (1997).

[b14] Brok B. D., Morel J. & Zahid M. In situ experimental study of roughness development at a stressed solid/fluid interface. Geol. Soc. 200, 73–83 (2002).

[b15] Tuffen H., Smith R. & Sammonds P. R. Evidence for seismogenic fracture of silicic magma. Nature 453, 511–514 (2008). 18497823 10.1038/nature06989

[b16] Silva P. F. *et al.* Magma flow, exsolution processes and rock metasomatism in the Great Messejana-Plasencia dyke (Iberian Peninsula). Geophys. J. Int. 175, 806–824 (2008).

[b17] Lavallee Y. *et al.* Seismogenic lavas and explosive eruption forecasting. Nature 453, 507–510 (2008). 18497822 10.1038/nature06980

[b18] Byrne J. Influence of sub-surface defects on low-cycle fatigue life in a gas turbine disc alloy at elevated temperature. Int. J. Fatigue 21, 195–206 (1999).

[b19] Hermann R., Birley S. S. & Holdway P. Liquation cracking in aluminium alloy welds. Mater. Sci. Eng. A 212, 247–255 (1996).

[b20] Robinson J. L. & Scott M. H. Liquation cracking during the welding of austenitic stainless steels and nickel alloys. Philos. Trans. R. Soc. London. Ser. A, Math. Phys. Sci 295, 105–117 (1980).

[b21] Montazeri M. & Ghaini F. M. The liquation cracking behavior of IN738LC superalloy during low power Nd:YAG pulsed laser welding. Mater. Charact. 67, 65–73 (2012).

[b22] Farup I., Drezet J. & Rappaz M. *In situ* observation of hot tearing formation in succinonitrile-acetone. Acta Mater. 49, 1261–1269 (2001).

[b23] Eskin D. G., Suyitno, & Katgerman L. Mechanical properties in the semi-solid state and hot tearing of aluminium alloys. Prog. Mater. Sci. 49, 629–711 (2004).

[b24] Kumar P., Lakshmi H. & Dutta P. Solidification of A356 alloy in a linear electromagnetic stirrer. Solid State Phenom. 141-143, 563–568 (2008).

[b25] Flemings M. C. Behavior of metal alloys in the semisolid state. Metall. Trans. B 22, 269–293 (1991).

[b26] Ferrante M. & de Freitas E. Rheology and microstructural development of a Al–4 wt%Cu alloy in the semi-solid state. Mater. Sci. Eng. A 271, 172–180 (1999).

[b27] Dahle A. K. & Arnberg L. Development of strength in solidifying aluminium alloys. Acta Mater. 45, 547–559 (1997).

[b28] Martin C. L., Brown S. B., Favier D. & Surey M. Shear deformation of high solid fraction (>0.60) semi-solid Sn-Pb under various structures. *M*. *at*er. Sci. Eng. A 202, 112–122 (1995).

[b29] Bigot R., Favier V. & Rouff C. Characterisation of semi-solid material mechanical behaviour by indentation test. J. Mater. Process. Technol. 160, 43–53 (2005).

[b30] Doherty R. D., Lee H.-I. & Feest E. A. Microstructure of stir-cast metals. Mater. Sci. Eng. 65, 181–189 (1984).

[b31] Ji S. The fragmentation of primary dendrites during shearing in semisolid processing. J. Mater. Sci. 38, 1559–1564 (2003).

[b32] Ananiev S., Nikrityuk P. & Eckert K. Dendrite fragmentation by catastrophic elastic remelting. Acta Mater. 57, 657–665 (2009).

[b33] Swann P. R. Dislocation substructure vs transgranular stress corrosion susceptibility of single phase alloys. Corrosion 19, 102t–114t (1963).

[b34] Drakopoulos M. *et al.* I12: the Joint Engineering, Environment and Processing (JEEP) beamline at Diamond Light Source. J. Synchrotron Radiat. 22, 828–838 (2015). 25931103 10.1107/S1600577515003513 PMC4416690

[b35] Majmudar T. S. & Behringer R. P. Contact force measurements and stress-induced anisotropy in granular materials. Nature 435, 1079–1082 (2005). 15973358 10.1038/nature03805

[b36] Silbert L. E., Grest G. S. & Landry J. W. Statistics of the contact network in frictional and frictionless granular packings. Phys. Rev. E 66, 61303 (2002). 10.1103/PhysRevE.66.061303 12513276

[b37] Yuan L., O'Sullivan C. & Gourlay C. M. Exploring dendrite coherency with the discrete element method. Acta Mater. 60, 1334–1345 (2012).

[b38] Dexter A. R. & Tanner D. W. Packing densities of Mixtures of Spheres with Log-normal Size Distributions. Nat. Phys. Sci. 238, 31–32 (1972).

[b39] Fallet A., Chichignoud G., Martin C. L., Suéry M. & Jarry P. Influence of barium addition on the microstructure and the rheological behaviour of partially solidified Al–Cu alloys. Mater. Sci. Eng. A 426, 187–193 (2006).

[b40] Joseph B., Picat M. & Barbier F. Liquid metal embrittlement: A state-of-the-art appraisal. Eur. Phys. J. Appl. Phys. 5, 19–31 (1999).

[b41] Phillion A. B. *et al.* *In situ* X-ray observation of semi-solid deformation and failure in Al–Cu alloys. Acta Mater. 59, 1436–1444 (2011).

[b42] Asaro R. J. & Tiller W. A. Interface morphology development during stress corrosion cracking: Part I. Via surface diffusion. Metall. Trans. 3, 1789–1796 (1972).

[b43] Koehn D., Arnold J., Jamtveit B. & Malthe-sørenssen A. Instabilities In Stress Corrosion And The Transition To Brittle Failure. Am. J. Sci. 303, 956–971 (2003).

[b44] Rice J. R. On the Structure of Stress-Strain Relations for Time-Dependent Plastic Deformation in Metals. J. Appl. Mech. 37, 728–737 (1970).

[b45] Asaro R. J., Tiller W. A. & Univer S. Interface morphology development during stress corrosion cracking: Part I. Via surface diffusion. Metall. Trans. 3, 1789–1796 (1972).

[b46] Puncreobutr C. *et al.* Influence of Fe-rich intermetallics on solidification defects in Al–Si–Cu alloys. Acta Mater. 68, 42–51 (2014).

[b47] Cai B. *et al.* Time-resolved synchrotron tomographic quantification of deformation-induced flow in a semi-solid equiaxed dendritic Al–Cu alloy. Scr. Mater. 103, 69–72 (2015).

[b48] Bower A. F., Fleck N. A., Needleman A. & Ogbonna N. Indentation of a power law creeping solid. Proc. R. Soc. Lond. A 441, 97–124 (1993).

[b49] Stampanoni M. *et al.* Trends in synchrotron-based tomographic imaging: the SLS experience. Proc. SPIE 63180M (2006).

[b50] Fife J. L. *et al.* Development of a laser-based heating system for *in situ* synchrotron-based X-ray tomographic microscopy. J. Synchrotron Radiat. 19, 352–358 (2012). 22514169 10.1107/S0909049512003287 PMC3329956

[b51] Titarenko S., Titarenko V., Kyrieleis A. & Withers P. J. A ring artifact suppression algorithm based on a priori information. Appl. Phys. Lett. 95, 071113 (2009).

