# Supplementary material for: Transgranular liquation cracking of grains in the semi-solid state
Source: Nat Commun. 2015 Sep 10;6:8300. doi: 10.1038/ncomms9300 (PMC4579839; doi:10.1038/ncomms9300)
Supplement: Supplementary Information — Supplementary Figures 1-4, Supplementary Table 1 and Supplementary Note 1 [file ncomms9300-s1.pdf]

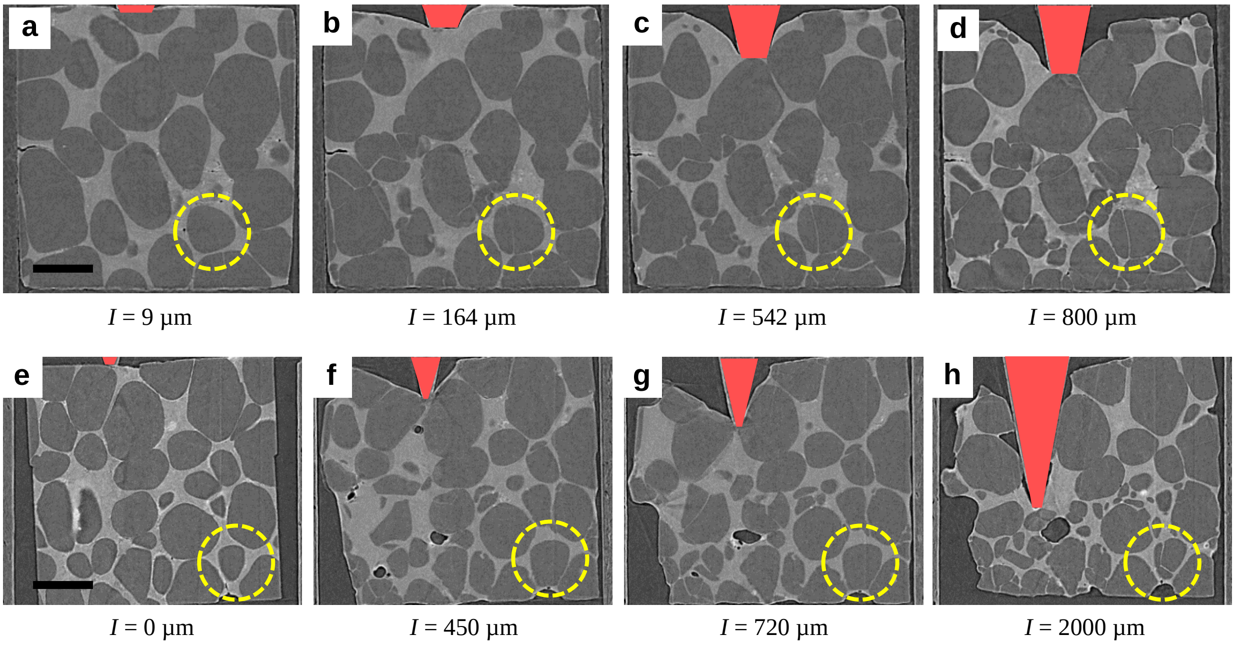

Supplementary Figure 1 | Semi-solid indentation at different deformation rates. 2D longitudinal sections showing a sequence of cracking during semi-solid indentation of an Al-15 wt.%Cu alloy at (a-d) 0.5 µm s^{-1} and (e-h) 10 µm s^{-1}. '*I*' represents the position of the indenter relative to its start position (Scale bar 600 µm). Note that in these cases, the region of prominent cracking is below the indenter (shown in dashed yellow circles). Note also that in case (i), the grain structure is perturbed as the indenter directly encounters a grain, whereas in (ii), the indenter enters the sample through the intergranular region.

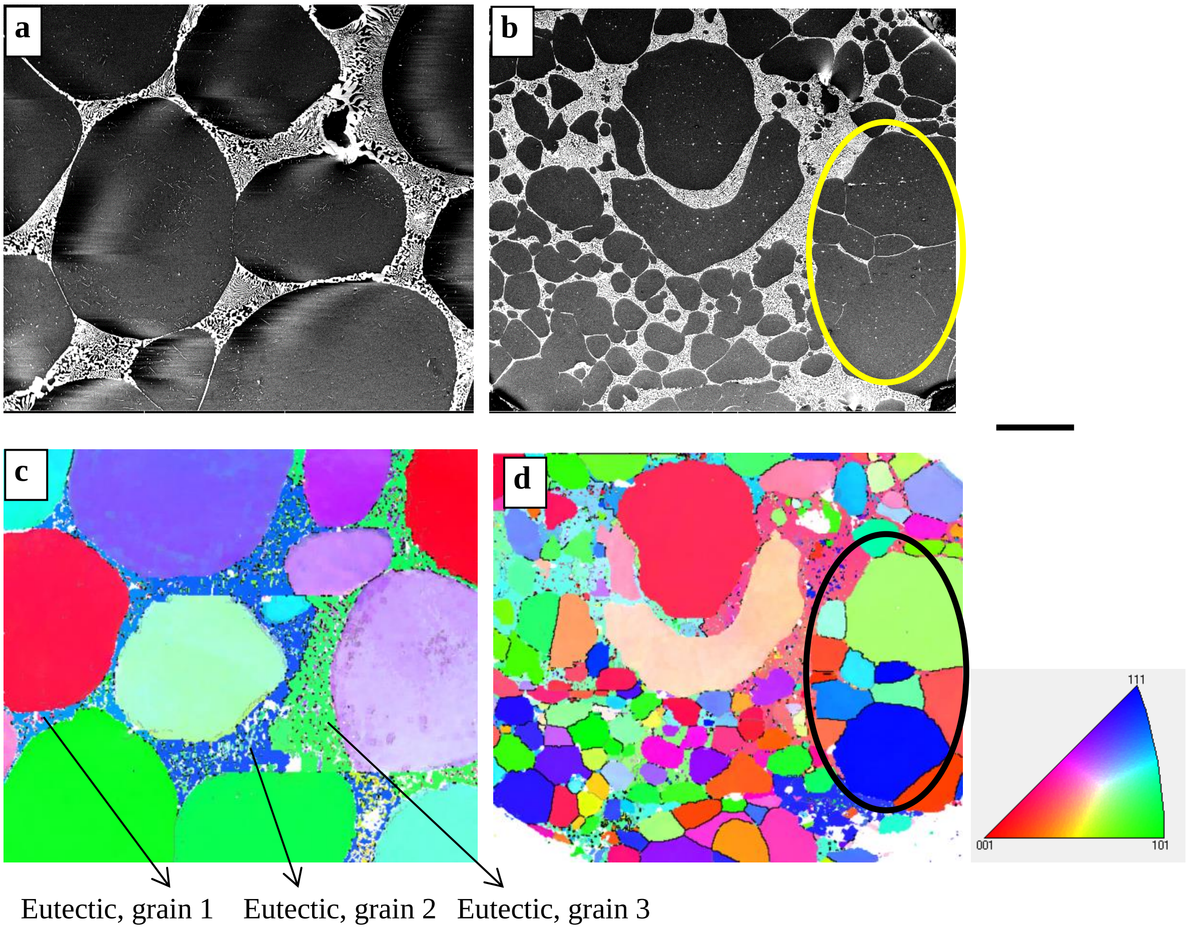

Supplementary Figure 2 | Evidence of transgranular cracking. SEM micrographs of the (a) initial sample, and (b) the cracked final specimen (after indentation at 2 µm s^{-1} at 555 °C). The circled region shows presence of liquid films which are not detected by the synchrotron images due to limited resolution. EBSD orientation maps (Euler colours) of the (c) initial specimen exhibiting a coarse grain structure, and (d) the cracked specimen showing fragmented grains separated by eutectic which was liquid at the cracking temperature (after indentation at 2 µm s^{-1} at 555 °C). In the as-cast structure (on a representative randomly selected sample slice) there are three eutectic grains, grain 1 and 2 with orientations in vicinity of [111] and similar the grain 2 is oriented with its [101] axis parallel to the sample cylinder axis. The rest of the microstructure comprises of α grains with a fairly random texture. (Scale bar 250 µm). See supplementary note 1 for further details.

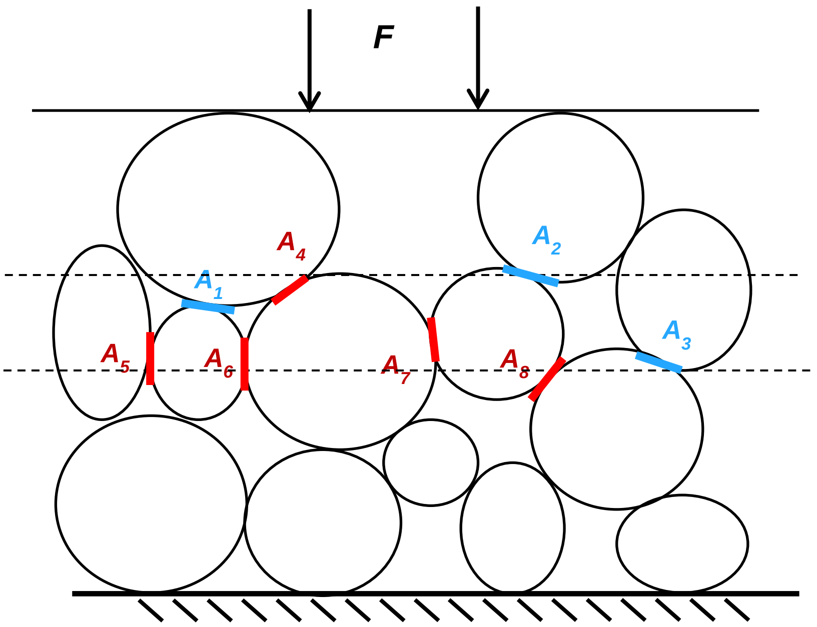

Supplementary Figure 3 | Estimation of contact stresses. This figure is a schematic diagram, showing how the contact stress was estimated, with lower and upper bounds. Areas represented in blue indicate contact areas normal to the applied load direction, which are likely to directly transfer the load, whereas those in red represent all other contact areas within a layer of a constrained grains. Note that this representation is shown in 2D only to give a simplified explanation of the process, but the calculations are performed on a 3D volume, by evaluating the orientation of contact areas using Avizo®. From the tomographic datasets, calculated areas are $\sum_{k=1}^{normal} A_k$ = 42087 μm^2, $\sum_{k=1}^{all} A_k$ = 443186 μm^2 and A_1 = 12997 μm^2.

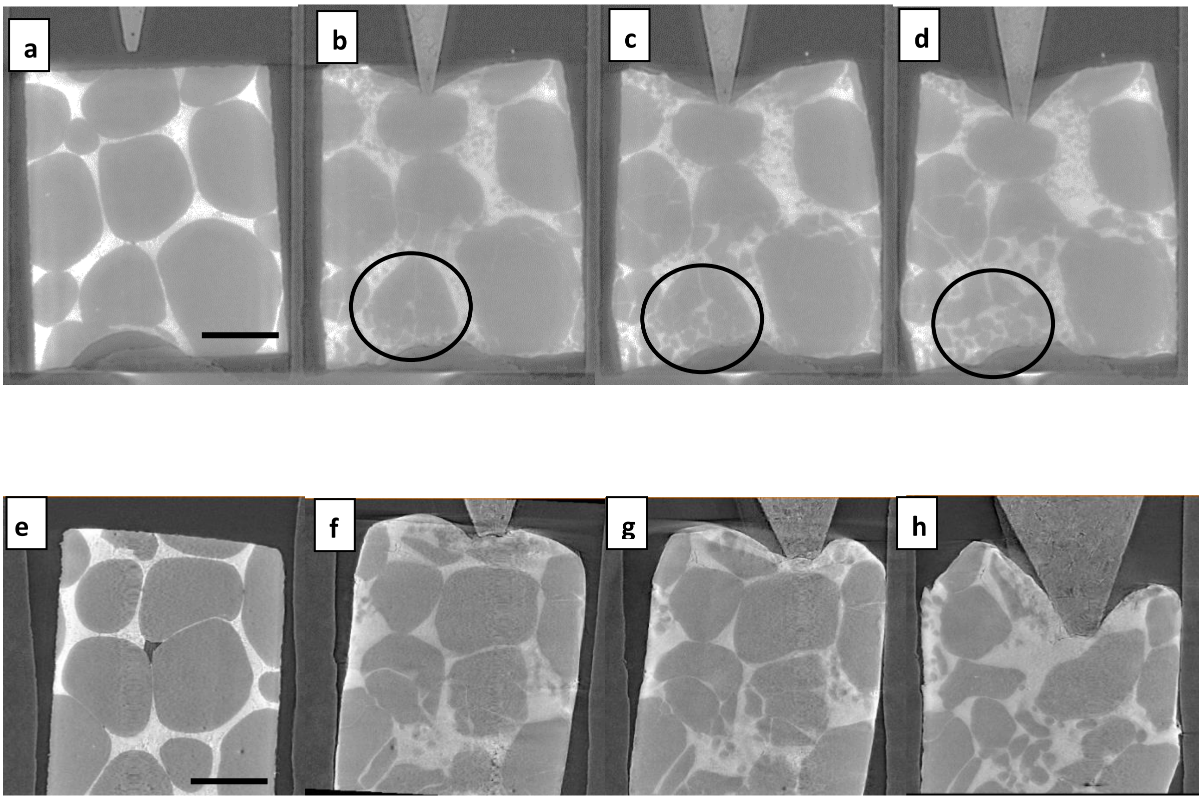

Supplementary Figure 4 | Influence of indenter shapes on TLC. Sequence of images from the SLS experiment using 1.7 µm pixel size and showing similar cracking behavior during indentation at 74% fraction solid. (a-d) Conical indenter (15° cone angle) moving at 2 µm s^{-1} speed, (e-h) larger square-pyramidal indenter (45° vertex angle) moving at 5 µm s^{-1} speed. Both the samples are 1.8 mm in diameter. (Scale bar 500 µm). The experiment was also repeated with a different heating source and indenter shapes at the TOMCAT beamline of the Swiss Light Source (SLS). Similar cracking behaviour was observed, and these results confirm that the mechanism is independent of the type of indenter and heating source (Supplementary Fig. 4); thus, it is due to constrained granular packing and deformation over a length scale of an average grain. Note the recursive nature of the grain fragmentation in figures b-d (shown in the black circled region), where the grain and its subsequent fragments continue to be stressed beyond the upper bound.

Supplementary Table 1 | Details of the experiments performed at both Diamond (DLS) and Swiss (SLS) Light Sources. The experiments at the TOMCAT beamline of SLS were performed with a smaller pixel size and thus used a smaller specimen.

Experiment details	Temperature [^{o}C]	Deformation speed [µm s^{-1}]	Cracking?
I12 Beamline, DLS<br>Beam: Monochromatic beam 53 keV<br>Pixel size: 4 µm/pixel<br>Sample size: ∅ 3 × 3 mm cylinder<br>Heating: Resistance furnace	555	0.5	Yes (Supplementary Fig. 1)
		2	Yes (Fig. 1, Supplementary Movie 1)
		10	Yes (Supplementary Fig. 1)
TOMCAT Beamline, SLS<br>Beam: Polychromatic beam with 5% filter, peak energy: ~30 keV<br>Pixel size: 1.7 µm/pixel<br>Sample size: ∅ 1.8 × 2 mm cylinder<br>Heating: Laser furnace	555	2, 5	Yes (Supplementary Fig. 4) (with two types of indenters)
	570	0.5	No (Fig. 4)

Supplementary Note 1:

SEM and EBSD were performed on as-cast and cracked specimen using a JSM-6610LV microscope to verify the composition and possible presence of oxide particles (Supplementary Fig. 2). No oxide particles were detected within the globular grains. However, Cu-rich pockets of a few microns in size can be observed, which can act as potential strain accumulation sites. Also note the presence of thin liquid films in the cracked grains (circled region in Supplementary Fig. 2b) that are not captured in the synchrotron images due to limited resolution.

Fig. c-d show EBSD images of the initial structure and final structures (of different samples as EBSD is destructive) with the orientation map. Each primary grain in the as-cast sample is a single-crystal with no internal high angle grain boundaries. In the cracked specimen the fragmented grains have both high and low angle GBs (separated by black and white lines respectively). This transformation in crystal orientation for a single initial grain can be seen in the circled region in Supplementary Fig. 2b, which originated from a single parent grain. However, the orientation of the fragments (Supplementary Fig. 2d) are different, hypothesised to be due to rotation of grains under continued indentation, with some pinned together by friction (low angle) and others rotating significantly due to granular flow under the large strains. Examining the grain in the centre of Supplementary Fig. 2d we see a fragment that looks like a smiley face – here the rotation is small since it is a ball in a cup. Interestingly, the EBSD images also show that the eutectic phase has formed large, equiaxed grains as well that do not seem to show an obvious crystallographic correlation to the primary phase in this slice.
